# Assessment of Acute Oral and Dermal Toxicity of 2 Ethyl-Carbamates with Activity against *Rhipicephalus microplus* in Rats

**DOI:** 10.1155/2014/956456

**Published:** 2014-05-06

**Authors:** María Guadalupe Prado-Ochoa, Ricardo Alfonso Gutiérrez-Amezquita, Víctor Hugo Abrego-Reyes, Ana María Velázquez-Sánchez, Marco Antonio Muñoz-Guzmán, Patricia Ramírez-Noguera, Enrique Angeles, Fernando Alba-Hurtado

**Affiliations:** ^1^Programa de Maestría y Doctorado en Ciencias de la Producción y de la Salud Animal, Universidad Nacional Autónoma de México, 04510 Ciudad Universitaria, DF, Mexico; ^2^Laboratorio de Química Medicinal, Departamento de Ciencias Químicas, Facultad de Estudios Superiores Cuautitlán, Universidad Nacional Autónoma de México, 54714 Cuautitlán Izcalli, MEX, Mexico; ^3^Departamento de Ciencias Biológicas, Facultad de Estudios Superiores Cuautitlán, Universidad Nacional Autónoma de México, 54714 Cuautitlán Izcalli, MEX, Mexico

## Abstract

The acute oral and dermal toxicity of two new ethyl-carbamates (ethyl-4-bromophenyl-carbamate and ethyl-4-chlorophenyl-carbamate) with ixodicide activity was determined in rats. The oral LD_50_ of each carbamate was 300 to 2000 mg/kg, and the dermal LD_50_ of each carbamate was >5000 mg/kg. Clinically, the surviving rats that had received oral doses of each carbamate showed decreased weight gain (*P* < 0.05) and had slight nervous system manifestations. These clinical signs were evident from the 300 mg/kg dose and were reversible, whereas the 2000 mg/kg dose caused severe damage and either caused their death or was motive for euthanasia. At necropsy, these rats had dilated stomachs and cecums with diffuse congestion, as well as moderate congestion of the liver. Histologically, the liver showed slight degenerative lesions, binucleated hepatocytes, focal coagulative necrosis, and congestion areas; the severity of the lesions increased with dosage. Furthermore, an slight increase in gamma-glutamyltransferase, lactate dehydrogenase, and creatinine was observed in the plasma. The dermal application of the maximum dose (5000 mg/kg) of each carbamate did not cause clinical manifestations or liver and skin alterations. This finding demonstrates that the carbamates under study have a low oral hazard and low acute dermal toxicity.

## 1. Introduction

*Rhipicephalus microplus* is the most important tick in tropical and subtropical areas in Mexico and throughout the world, causing great economic losses in livestock production [1]. For many years, the most used strategy for controlling ticks has been the use of chemical ixodicides. Nevertheless, the high selection pressure caused by their exaggerated use has promoted resistance to the main commercial ixodicides [2]. This resistance has compelled the development of new pharmaceutical alternatives for the control of ticks. Among these alternatives is the development of new molecules for which ticks have not developed resistance.

Our group has shown that the new carbamates synthesized in FES-Cuautitlan-UNAM, namely, ethyl-4-bromophenyl-carbamate (LQM 919) and ethyl-4-chlorophenyl-carbamate (LQM 996), negatively affect* R. microplus* biological parameters and reproduction, both in susceptible strains and in those resistant to the commercial ixodicides used in México [3, 4]. These carbamates caused alterations in the reproductive organs, vitellogenesis, and the viability of the ovarian cells, and these effects were found to be independent of acetylcholinesterase inhibition [5].

Before these new carbamates can be considered for use in the control of ticks it is necessary to assess the adverse effects that they could cause in mammals. Previous studies have shown that the toxicity of known carbamates is variable [6]. Some carbamates are highly toxic, for example aldicarb (2-methyl-2-[methylthio] propionaldehyde o-[methylcarbamoyl] Oxime) which has an oral 50% lethal dose (LD_50_) of 0.3 to 0.9 mg/kg, carbofuran ( 2,3-dihydro-2,2-dimethyl-7-benzofuranylmethylcarbamate) which has an oral LD_50_ of 8 mg/kg, and carbaryl (1-naphthyl methylcarbamate) which has an oral LD_50_ of 12.5 mg/kg [7]. Other carbamates such as propoxur (2-isopropoxyphenyl methylcarbamate), which has an oral LD_50_ of 68 to 94 mg/kg and dermal LD_50_ of >2000 mg/kg [8], are considered to be of mid-level toxicity. In contrast, benzimidazoles show low toxicity [9]. Albendazole (5-[propylthio]-1H-benzimidazol-2-yl carbamic acid methyl ester) shows an LD_50_ of 1320–2400 mg/kg, whereas mebendazole (methyl 5-benzoyl-1H-benzimidazol-2-yl-carbamate) has an oral LD_50_ of 715 to 1434 mg/kg [10].

In bovines, the proposed administration pathway for the carbamates LQM 919 and 996 is dermal using aspersion or immersion baths. Nevertheless, the dermal pathway also represents the highest risk for human contact with ixodicide products. Furthermore, due to the grooming behavior in bovines, they could ingest the products used in baths. Taking into consideration the aforementioned, in this study, we determined the acute oral and dermal toxicity in rats caused by the administration of the two new ethyl-carbamates with inhibitory activity on the embryonic development of* R. microplus*.

## 2. Materials and Methods

### 2.1. Animals

Clinically healthy 7- to 8-week-old male Wistar rats weighing between 175 and 200 g were used. All animals were kept in groups of 5 individuals. The environmental temperature was maintained at 22 ± 2°C with a relative humidity between 30 and 70% and a 12 : 12 light : dark cycle. They were fed with commercial feed and water* ad libitum*. This study was approved by the Internal Committee for the Care of Experimental Animals of the Postgraduate Program of Animal Production and Health (UNAM, México). The experiments of this work were conducted in single trial and designed based on Guidelines 420 and 423 of the Organization for Economic Cooperation and Development (OECD).

### 2.2. Evaluated Carbamates

The carbamates used in this study were designed and synthesized at the Universidad Nacional Autónoma de México, using a benzimidazole molecule as the structural base. The carbamates were synthesized by reacting aryl- and alkylamines with sodium hydride and benzene diethyl carbonate, followed by column chromatography purification. Next, the products were recrystallized. The carbamates were structurally characterized through interpretations of their IR spectra, H^1^ and C^13^ nuclear magnetic resonance, and mass spectrometry [11].

Since the carbamates used in this study are insoluble in water, they were first dissolved in 1 mL of dimethyl sulfoxide (DMSO) and later diluted to 2 mL using corn oil for oral administration or with water for dermal administration. The chemical structure, nomenclature, molecular weight, and identification key of the studied carbamates are shown in Table 1.

### 2.3. Experimental Design for Acute Oral Toxicity

A total of 50 Wistar rats distributed in 10 groups of 5 rats each were used. Rats in groups 1, 2, 3, and 4 received 5, 50, 300 and 2000 mg/kg, respectively, of LQM 919 dissolved in DMSO and corn oil. Rats in groups 5, 6, 7, and 8 received 5, 50, 300 and 2000 mg/kg, respectively, of LQM 996 dissolved in DMSO and corn oil. Rats in group 9 received DMSO dissolved in corn oil whereas rats in group 10 only received corn oil (control groups). Treatments were administered in a single dose using an intragastric tube [12].

Animals were kept under observation from day 5 before treatment until 14 days after treatment (p.t.). Rats that survived were humanely euthanized on day 14 p.t. [13].

Necropsies were carried out at the time of death or euthanasia and any observed macroscopic alterations were recorded. Samples were also taken from the lung, brain, cerebellum, intestine, stomach, liver, kidney, heart, and muscle. The collected samples were fixed in 4% paraformaldehyde and processed using conventional techniques for histopathological study. Also, blood was collected at the time of death or euthanasia in order to measure biochemical parameters, and liver samples were also used to quantify thiobarbituric acid reactive substances (TBARS).

### 2.4. Experimental Design for Acute Dermal Toxicity

A total of 40 male Wistar rats distributed in 8 groups of 5 rats each were used. Rats in groups 1, 2, and 3 received topically 500, 2000 and 5000 mg/kg, respectively, of LQM 919 dissolved in DMSO and water. Rats in groups 4, 5, and 6 received topically 500, 2000 and 5000 mg/kg, respectively, of LQM 996 dissolved in DMSO and water. Rats in group 7 received topically DMSO and water, whereas rats in group 8 only received topical water (control groups). The backs of the rats were shaved 24 hours before the dermal application of the carbamates. At the time of treatment, the corresponding carbamate dose was applied on the intact skin of the rats and a semiocclusive patch was placed on the application site for an exposure period of 24 hours. At the end of said period, the patch was removed and the exposure area was washed with water in order to remove any product residues.

Animals were kept under observation from day 5 before treatment up until 14 days p.t. All rats were humanely euthanized on day 14 p.t. [13]. Necropsies were carried out at the time of death or euthanasia and observed macroscopic alterations were recorded. Samples from lung, brain, cerebellum, intestine, stomach, liver, kidney, heart, and muscle were also taken. The collected samples were fixed in 4% paraformaldehyde and processed using conventional techniques for histopathological study. Also, blood was collected at the time of death or euthanasia in order to measure biochemical parameters, as well as liver samples to quantify TBARS.

### 2.5. Clinical Observations

Rats from both experiments were clinically checked twice a day prior to treatment and then every hour during the first eight hours p.t. and three times a day during the rest of the experiment. Skin, mucosa, eyes, respiratory rate, nasal secretions, salivation, tremors and convulsions, changes in activity levels, posture, gait, sensory reaction to stimulus, and strange behaviors were systematically checked following the table proposed by Morton and Griffiths [14]. Furthermore, the presence of erythema and edema in the skin area exposed to carbamates was assessed in rats treated dermally following the scale proposed by Draize et al. [15]. The weights of all rats were recorded at 0, 7, and 14 days p.t.

### 2.6. Biochemical Tests

Plasma was obtained from the blood samples and frozen at −80°C until it was processed. Plasma levels of aspartate aminotransferase (AST), alanine aminotransferase (ALT), lactate dehydrogenase (LDH), and gamma-glutamyltransferase (GGT) were measured using the methods described by Reitman and Frankel [16], Wooton [17], and Szasz [18], respectively. The concentrations of total protein, albumin, and plasma creatinine were determined by the methods established by Westgard and Poquette [19], Gornall et al. [20], and Bartels et al. [21], respectively. The aforementioned determinations were carried out using commercial kits from BioSystems. In order to calculate globulin concentrations in the plasma, the value of albumin was subtracted from that of total protein. The albumin and globulin (A/G) ratio was calculated by dividing the concentration of albumin by the concentration of globulin. Cholinesterase activity (CHE) was determined using the colorimetric method described by Ellman et al. [22] using a commercial kit from Wiener Lab.

### 2.7. TBARS Quantification

Samples (0.5 cm^3^ in size) were collected from the liver of all rats, submerged in a buffer solution (PBS pH 7.2, 15 mM sodium azide, 1 mM PMSF, 0.1% Triton X-100, and 5 mM EDTA), and frozen in liquid nitrogen until further processing. Tissues were thawed on ice, and the original buffer solution was discarded. Then, recently prepared buffer solution was added and the tissues were then homogenized mechanically, sonicated (3 pulses of 10 seconds, 50% amplitude), and finally centrifuged (13000 g, 7′ to 4°C). The supernatant (40 *μ*L) was added to an equal volume of 2.5% perchloric acid and incubated at ambient temperature for 10 minutes. Afterwards, samples were centrifuged at 13000 g for 10 minutes at 4°C and the supernatant was reacted with 0.067% thiobarbituric acid (TBA) at 90°C for 30 minutes [23]. The TBARS content of the samples was quantified using a standard curve generated using malondialdehyde (MDA) at 532 nm [24]. The concentration of TBARS was expressed in nmol/mg of protein.

### 2.8. Statistical Analysis

The data regarding weight gain, plasma levels of AST, GGT, LDH, ALT, CHE, and creatinine, and TBARS concentrations in the liver were analyzed using a one-way ANOVA, and differences between the means were established with Fisher's post hoc tests (i.e., minimum significant differences) using the minimum confidence level of 95%.

## 3. Results

### 3.1. LD_50_

The number of rats who died or were euthanized due to clinical signs of severe toxicity due to the oral or dermal administration of the carbamates in this study is shown in Table 2. The LD_50_ for the oral pathway of each carbamate was found to be between 300 and 2000 mg/kg. Also, the LD_50_ for the dermal pathway of each carbamate was >5000 mg/kg.

### 3.2. Body Weight

The oral administration of 5 and 50 mg/kg of each carbamate did not have an effect on weight gain in treated rats (*P* > 0.05). Surviving rats that had received orally 300 mg/kg of carbamates LQM 919 and LQM 996 (39.7 ± 12.1 g and 23 ± 12.5 g, resp.) showed decreased weight gain (*P* < 0.01) when compared to rats in the control groups (corn oil + DMSO 88.6 ± 17.6 g; corn oil 55.6 ± 6.5 g). None of the dosages applied dermally had an effect on the weight gain (*P* > 0.05) of treated rats when compared to the control rats (water 52.2 ± 15 g; water + DMSO 51.8 ± 14.5 g).

### 3.3. Clinical Manifestations

The oral administration of 5 and 50 mg/kg of each carbamate did not produce clinical manifestations associated with toxicity in treated rats. The clinical manifestations observed in rats treated orally with 300 and 2000 mg/kg of each carbamate are shown in Table 3. None of the dosages of each carbamate administered dermally produced clinical manifestations associated with toxicity in rats.

### 3.4. Findings at Necropsy

Macroscopically, rats that died or that were euthanized after the oral administration of 2000 mg/kg of carbamate LQM 919 only exhibited a dilated stomach and cecum with diffuse congestion. The single rat that survived from this group until the end of the study had moderate congestion of the liver. Two of the rats that received 300 mg/kg and 4 of those that received 2000 mg/kg of carbamate LQM 996 had moderate diffuse congestion of the liver. The remainder of the rats that received the oral dosage together with all rats that received the dermal dosages and the control rats did not show apparent pathological changes at necropsy.

### 3.5. Histopathology

**Figure 1:**
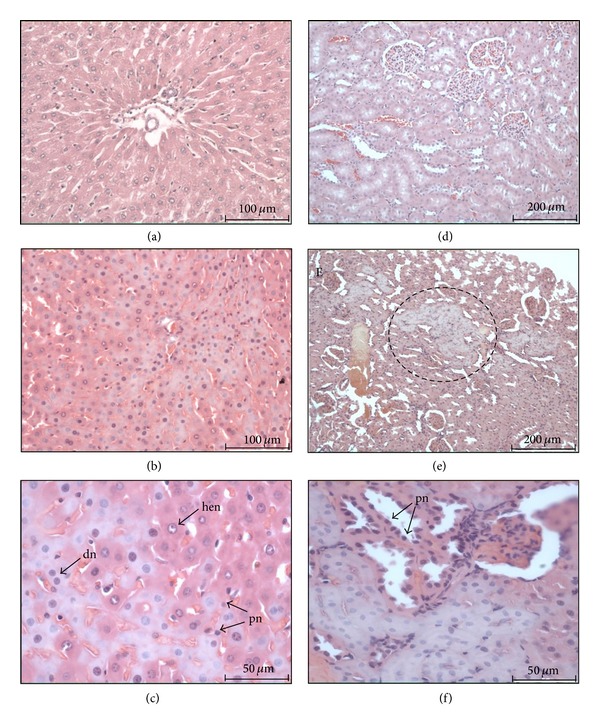
Histopathological findings in rats with oral exposure to carbamate LQM 919. (a) Liver section (20x) of an unexposed rat (control). (b) Liver section (20x) of a rat exposed to 2000 mg/kg of carbamate that shows a degenerated area. (c) Previous image magnification (40x) that shows highly euchromatic nuclei (hen), hepatocytes with two nuclei (dn), and pyknotic nuclei (pn). (d) Kidney section (10x) of an unexposed rat (control). (e) Kidney section (10x) of a rat exposed to 2000 mg/kg of carbamate that shows hyaline degenerated foci (discontinuous circle) in the renal cortex. (f) Previous image magnification (40x) that shows hyaline degeneration and pyknotic nuclei (coagulative necrosis).

Rats from all experimental groups that received oral dosages of carbamate LQM 919 exhibited albuminous degeneration of the liver with the presence of hepatocytes with highly euchromatic nuclei and slight vacuolar degeneration. Furthermore, rats that received the 300 mg/kg dose showed an increase in hepatocytes with two nuclei and increased liver congestion. Rats that received the 2000 mg/kg dose showed coagulative necrotic foci as well as congestion in their liver and had hyaline degeneration and pyknotic nuclei (coagulative necrosis) in areas of their renal cortices (Figure 1).

Rats from all experimental groups that received oral doses of carbamate LQM 996 had necrotic foci and highly euchromatic nuclei in their livers; the severity of the lesions increased with increased dosage.

The remainder of the organs in rats that received oral administration of each carbamate did not show microscopic lesions. The rats that received the dermal administration and the rats from control groups did not show apparent microscopic lesions in the organs that were sampled.

### 3.6. Biochemical Tests

Rats that received orally 300 mg/kg of carbamate LQM 919 had an increase (*P* < 0.05) of GGT (2.45 ± 1.87 U/L) at day 14 p.t. when compared to the control group that received corn oil + DMSO (1.06 ± 0.68 U/L). Rats that received orally 2000 mg/kg of said carbamate showed between 3 and 22 hours p.t. (time of death or euthanasia) a higher concentration (*P* < 0.001) of LDH (1170 ± 120.6 U/L) when compared to the control group that received corn oil + DMSO (493.4 ± 114.2 U/L). The remainder of the rats that received dosages of carbamate LQM 919 either orally or dermally did not show significant differences (*P* > 0.05) in any of the biochemical parameters evaluated when compared to the control group (Tables 4 and 5).

Rats that received orally 300 mg/kg of carbamate LQM 996 had an increase (*P* < 0.001) in creatinine concentration (1.13 ± 0.64 U/L) at day 14 p.t. when compared to the control group that received corn oil + DMSO (0.49 ± 0.12 mg/dL). Rats that received dermally 5000 mg/kg of said carbamate showed a greater concentration (*P* < 0.05) of LDH (1270 ± 652.2 U/L) when compared to the control group that received corn oil + DMSO (603.0 ± 172.1 U/L). The remainder of the rats that received dosages of carbamate LQM 996 either orally or dermally did not show differences in any of the biochemical parameters evaluated when compared to the control group (Tables 6 and 7).

### 3.7. TBARS

**Figure 2:**
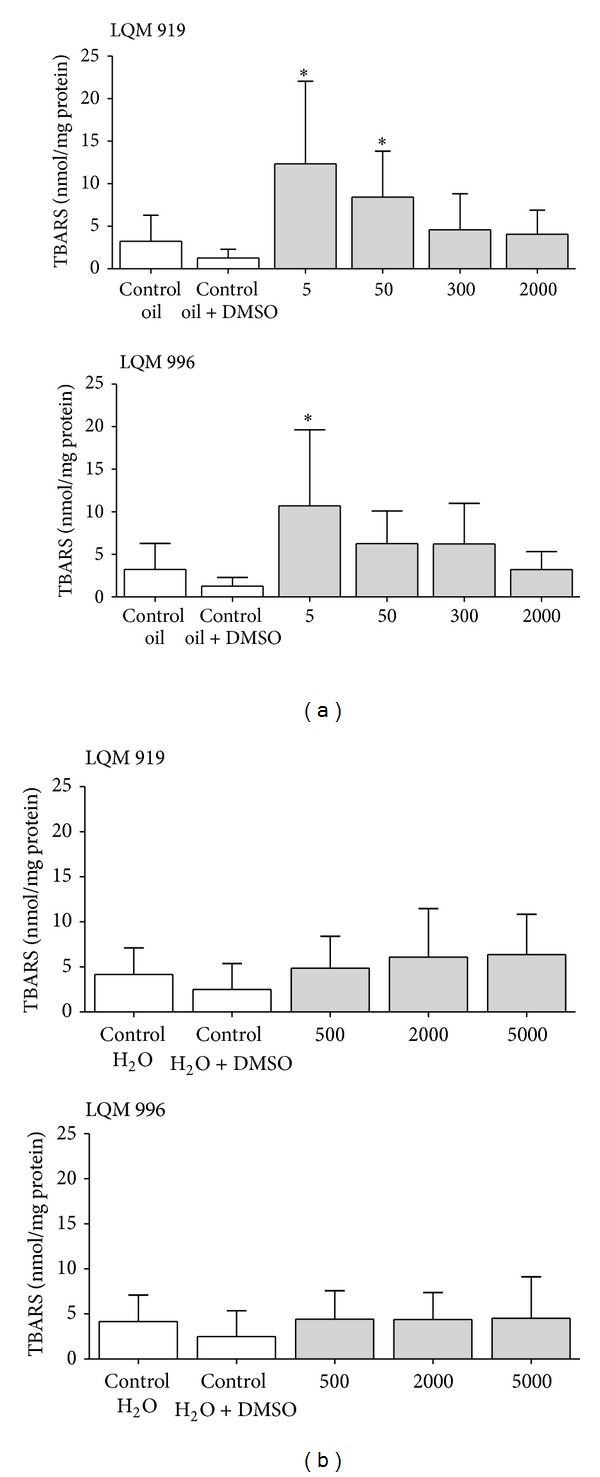
Mean (±SD) levels of thiobarbituric reactive substances (TBARS) in the livers of Wistar rats orally (a) or dermally (b) exposed to different concentrations of the carbamates LQM 919 or LQM 996. *Significant difference with the control group (*P* < 0.05).

Rats in the groups that received oral dosages of 5 or 50 mg/kg of carbamate LQM 919 (12.3 ± 9.7 and 8.4 ± 5.4 nmol/mg, resp.) and those that received the 5 mg/kg dosage of carbamate LQM 996 (10.7 ± 8.9 nmol/mg) showed an increase (*P* < 0.05) in the concentration of TBARS in the liver when compared to the control rats that received corn oil + DMSO (1.2 ± 1.0 nmol/mg). Rats in the remaining groups that received dosages of each carbamate did not show significant differences (*P* > 0.05) when compared to the control groups. Rats of all groups that received a dermal administration of each carbamate did not show differences (*P* > 0.05) with the control groups (Figure 2).

## 4. Discussion

A desirable characteristic of any drug is that it has therapeutic effects at low dosages and has the least amount of undesirable secondary and toxic effects on individuals. Previous work has shown that the synthetic carbamates that were evaluated in this study inhibit the* in vitro* reproduction of* R. microplus* at low concentrations [3]. The results of this study, following the guidelines [12] of the OECD, show that the synthetic carbamates evaluated in this study have low toxicity in rats.

The LD_50_ is used as a general indicator of acute toxicity of a substance. Currently, the majority of studies conducted to evaluate the acute toxicity of new pharmaceuticals is based on the Fixed Dose Procedure recommended by the OECD [12], so this study used said methodology. The oral pathway LD_50_ of both carbamates was 300 to 2000 mg/kg. Taking into account the criteria set by the Globally Harmonized System (GHS) of Classification and Labeling of Chemicals [25], these carbamates can be classified as category 4 (low hazard). Other pesticides that are considered by the GHS to have high oral toxicity such as coumaphos (LD_50_ 13 mg/kg) or be moderately toxic such as diazinon, chlorpyriphos, and malathion (LD_50_ 76 mg/kg, 82 mg/kg and 290 mg/kg, resp.) are currently being used commercially [9]. In this context, the estimation of the higher LD_50_ of the carbamates evaluated in this study suggests a relative reduced acute hazard in mammals.

The effects of the carbamates evaluated in this study on engorged female ticks in Adult Immersion Tests suggest that they should be used directly on the bovine's skin [3]. The dermal application of up to 5000 mg/kg of the carbamates in this study did not cause death in any of the treated rats (dermal LD_50_ > 5000 mg/kg). Therefore, according to the GHS criteria they should be classified as category 5 (low acute toxicity). The data show that the carbamates evaluated in this study have less dermal toxicity than has been reported in the Pan Pesticide Database for other commercial pesticides (coumaphos, dermal LD_50_ of 860 mg/kg; diazinon, dermal LD_50_ of 455 mg/kg; chlorpyriphos, dermal LD_50_ of 202 mg/kg; and malathion, dermal LD_50_ of 4444 mg/kg).

The safety margin of an ixodicide can be estimated indirectly by calculating the relationship between the oral or dermal LD_50_ and the effective concentration on ticks. The ratio between the oral LD_50_ (<300 mg/kg) or dermal LD_50_ (>5000 mg/kg) levels in rats obtained in this study and the concentration that inhibits tick reproduction that has been previously reported (LQM 919 = 0.687 mg/mL and LQM 996 = 0.279 mg/mL; [3]) were 436:1 and 7278 : 1 for LQM 919 (oral and dermal, resp.) and 1075 : 1 and 17921 : 1 for LQM 996 (oral and dermal, resp.). If this same calculation method is used for the data found in the literature [9, 26], then coumaphos has a ratio of 65 : 1 and 4300 : 1 (oral and dermal, resp.) and chlorpyriphos has a ratio of 273 : 1 and 673 : 1 (oral and dermal, resp.). These data suggest that the carbamates evaluated in this study have a greater safety range than some organophosphates that are currently being used commercially.

The oral administration of high doses (300–2000 mg/kg) of the studied carbamates caused in rats various levels of immobility, prostration, hypothermia, depression of spontaneous and provoked behavior, and paralysis with extension of hind quarters. These signs were evident from the 300 mg/kg dose and were reversible, whereas those that occurred at the 2000 mg/kg dose were severe and either caused their death or were the reason for euthanasia of the rats. The observed clinical signs coincide with the effects caused by other carbamates that reversibly inhibit acetylcholinesterase in the nervous system and cause the accumulation of acetylcholine in cholinergic synapses. Nevertheless, plasma cholinesterase levels did not decrease in treated rats. Furthermore,* in vitro *observations made by our group showed that the carbamates used in this study are weak inhibitors with a low affinity for acetylcholinesterase in* R. microplus* [5]. In light of this finding, we associate the clinical signs observed in treated rats with the weak inhibitor effect on acetylcholinesterase of the carbamates that were evaluated in this study.

The body weight gain of experimental animals is an indicator of the degree of wellness and health of the subjects. The surviving rats at the oral dosage of 300 and 2000 mg/kg of carbamates evaluated in this study exhibited decreased weight gain (*P* < 0.05) when compared to rats in the control group. This finding indicates that high oral dosages of the carbamates have an effect on the general wellbeing of the treated rats. In contrast, their dermal application did not have an effect on the weight gain or general condition of rats.

Some enzymes such as AST, ALT, GGT, and LDH are used as bioindicators of liver damage. An elevation in their plasma levels is mainly due to the alterations in the hepatocyte membrane or changes in its permeability [27]. The carbamates that were studied caused a moderate elevation of GGT in rats that received a 300 mg/kg oral dose. The increase in GGT levels indicates that there was damage to hepatocytes, although the absence of increases of the other transaminases suggests that the damage is minor. The enzyme GGT has an important role in antioxidant homeostasis and is frequently upregulated after acute oxidative stress. There is some evidence of the elevation in systemic GGT activity, which is characterized by the extended generation of reactive oxygen species [28, 29]. In this work, we observed an increase of TBARS in the livers of rats treated orally with the evaluated carbamates. These results suggest the association of toxic effects in rats exposed orally and oxidative damage estimated by the increase in TBARS. More studies are necessary in order to elucidate the action mechanism of the new carbamates and its likely effect on the modulation of oxidative stress. Histologically, they caused liver damage, and the severity of the observed lesions was dose-dependent. Rats that died during the first 24 hours p.t. did not show histological changes in their livers. In contrast, rats that were euthanized at 14 days p.t. had various levels of degenerative and necrotic processes. Taking into consideration our results, we conclude that the carbamates evaluated in this study have a moderate hepatotoxic effect that is manifested several days after treatment. Further studies are needed to assess the chronic and subchronic damage on the liver caused by these carbamates in order to fully determine their toxicity.

The kidneys are organs that purify toxic substances and are frequently affected by the toxic action of some compounds [30] An increase in the plasma levels of creatinine and the presence of lesions that suggest coagulative necrosis in the renal cortex of rats treated with high doses of the carbamates in this study indicate a slight nephrotoxic effect.

One of the most important factors in determining the toxicity of a drug is its rate of absorption, which generally varies according to the administration pathway. In this study, although the plasma levels of the carbamates were not measured, higher amounts of toxic effects (clinical signs, lesions, and functional alterations) were observed in rats treated orally than in those treated dermally. This finding suggests that these compounds are well absorbed in the digestive tract but have limited absorption through the skin. Thus, it is important that future studies evaluate the absorption, distribution, metabolism, and excretion of these carbamates.

## 5. Conclusion

The results obtained in this study indicate that both carbamates administered orally in a single dose are low hazard. Nevertheless, there are signs of toxicity at the higher dosages, which will allow us to determine the dosage range that can be used in future chronic and subchronic toxicity studies. The probable administration pathway of these compounds for tick control is dermal. The low dermal acute toxicity and the absence of erythema, edema, or corrosion on the skin of rats that were exposed to these carbamates make it possible to test them on bovines in order to evaluate their efficacy in the control of the cattle tick* R. microplus.*

## Figures and Tables

**Table 1:** Chemical structures and molecular weights of the evaluated carbamates.

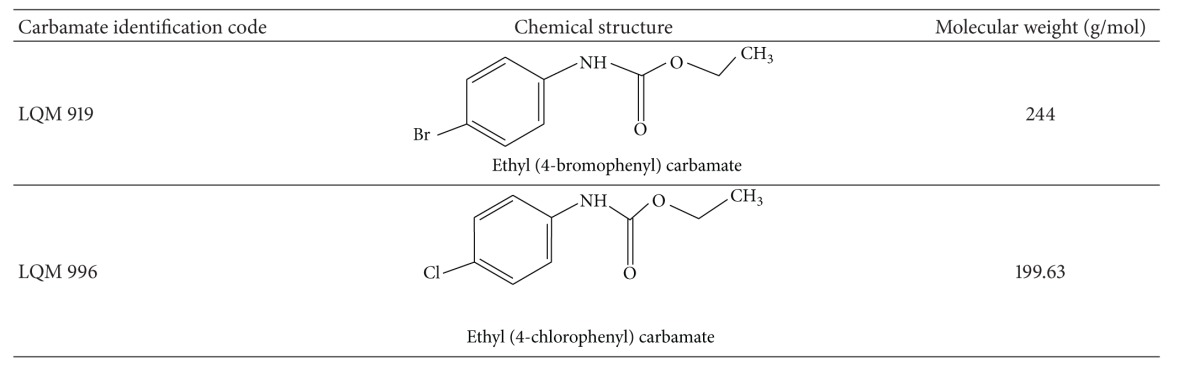

**Table 2:** Mortality of rats treated and untreated with two new carbamates administered by oral or dermal pathway.

Treatment		LQM 919	LQM 996	Control (corn oil or water)	Control (corn oil or water + DMSO)
Administration pathway	Dose (mg/kg)	Mortality (*n* = 5)			
Oral	0	—	—	0	0
	5	0	0	—	—
	50	0	0	—	—
	300	1	0	—	—
	2000	4	5	—	—

Dermal	0	—	—	0	0
	500	0	0	—	—
	2000	0	0	—	—
	5000	0	0	—	—

**Table 3:** Onset and evolution of clinical manifestations observed in rats orally exposed to carbamates LQM 919 or LQM 996.

Treatment	Dose (mg/kg b.wt.)	Number of rats (*n* = 5)	Clinical signs	Onset of clinical signs (hours p.t.)	Recovery time (hours p.t.)	Time of death or euthanasia (hours p.t.)
LQM 919	300	1	Hypotensive shock	0.016		Death 0.016
		4	Mild depression in unprovoked and provoked behavior	1	8 to 24	
	2000	1	Weakness and prostration	1	8 to 24	
		1	Weakness, prostration, and hypothermia	1		Death 3
		3		1		Euthanasia 8–22

LQM 996	300	5	Mild depression in unprovoked and provoked behavior	1	8 to 24	
	2000	3	Weakness, prostration, and hypothermia	1		Euthanasia 9
		2		1		Death 5

p.t.: posttreatment.

**Table 4:** Effect of the acute oral exposure of LQM 919 on selected parameters (mean ± SD) in Wistar rats.

Parameter (units)	Group					
	Control (corn oil)	Control (corn oil + DMSO)	5 mg/kg	50 mg/kg	300 mg/kg	2000 mg/kg
AST (U/L)	197.6 ± 72.89	162.3 ± 46.69	217.2 ± 90.81	198.2 ± 69.24	161.1 ± 23.12	215.7 ± 59.11
ALT (U/L)	66.92 ± 14.34	68.64 ± 23.14	74.26 ± 11.06	84.62 ± 17.99	89.54 ± 11.03	51.04 ± 11.03
LDH (U/L)	683.8 ± 164.1	493.4 ± 114.2	926.4 ± 156.8	692.0 ± 178.6	488.0 ± 119.1	1170 ± 120.6*
GGT (U/L)	1.27 ± 0.47	1.06 ± 0.68	0.70 ± 0.42	0.27 ± 0.26	2.45 ± 1.87*	1.86 ± 0.43
CHE (U/L)	315.4 ± 56.9	324.3 ± 82.1	310.9 ± 125.6	377.6 ± 95.5	288.7 ± 27.2	364.2 ± 41.1
Creatinine (mg/dL)	0.44 ± 0.03	0.49 ± 0.12	0.53 ± 0.04	0.50 ± 0.02	0.61 ± 0.12	0.48 ± 0.12
Total protein (g/dL)	6.30 ± 0.99	5.87 ± 0.86	5.79 ± 0.35	5.85 ± 0.55	6.05 ± 0.34	5.65 ± 0.57
Albumin (g/dL)	2.04 ± 0.78	2.57 ± 0.73	2.61 ± 0.60	2.51 ± 0.62	2.21 ± 0.78	2.46 ± 0.76
Globulin (g/dL)	4.26 ± 0.92	3.31 ± 0.75	3.17 ± 0.32	3.34 ± 0.71	3.84 ± 1.09	3.18 ± 1.16
A/G	0.52 ± 0.29	0.81 ± 0.30	0.84 ± 0.25	0.79 ± 0.29	0.66 ± 0.36	0.91 ± 0.47

AST: aspartate aminotransferase; ALT: alanine aminotransferase; LDH: lactate dehydrogenase; GGT: gamma-glutamyltransferase; CHE: cholinesterase; A/G: albumin and globulin. *Significant difference with the control group (*P* < 0.05).

**Table 5:** Effect of the acute dermal exposure of LQM 919 on selected parameters (mean ± SD) in Wistar rats.

Parameter	Group				
	Control (corn oil)	Control (corn oil + DMSO)	500 mg/kg	2000 mg/kg	5000 mg/kg
AST (U/L)	201.5 ± 62.90	218.6 ± 79.04	206.4 ± 34.56	171.6 ± 29.33	171.8 ± 31.37
ALT (U/L)	85.87 ± 9.81	86.47 ± 17.24	56.04 ± 26.57	64.86 ± 14.17	84.36 ± 9.71
LDH (U/L)	732.0 ± 129.5	603.0 ± 172.1	643.2 ± 164.6	611.6 ± 198.0	693.2 ± 108.4
GGT (U/L)	1.01 ± 0.51	1.15 ± 1.62	0.18 ± 0.25	0.47 ± 0.50	1.51 ± 0.83
CHE (U/L)	457.5 ± 161.4	360.9 ± 66.32	450.5 ± 114.3	408.7 ± 108.4	462.0 ± 64.06
Creatinine (mg/dL)	0.51 ± 0.23	0.64 ± 0.21	0.59 ± 0.16	0.55 ± 0.16	0.46 ± 0.09
Total protein (g/dL)	6.59 ± 0.45	6.38 ± 0.26	6.13 ± 0.71	6.69 ± 0.62	6.50 ± 0.49
Albumin (g/dL)	2.53 ± 0.99	2.51 ± 0.82	2.66 ± 0.54	2.72 ± 0.75	2.41 ± 0.69
Globulin (g/dL)	4.06 ± 1.20	3.87 ± 1.03	3.48 ± 0.62	3.97 ± 0.18	4.09 ± 0.92
A/G	0.72 ± 0.41	0.73 ± 0.40	0.79 ± 0.21	0.69 ± 0.21	0.64 ± 0.30

AST: aspartate aminotransferase; ALT: alanine aminotransferase; LDH: lactate dehydrogenase; GGT: gamma-glutamyltransferase; CHE: cholinesterase; A/G: albumin and globulin.

**Table 6:** Effect of the acute oral exposure of LQM 996 on selected parameters (mean ± SD) in Wistar rats.

Parameter	Group					
	Control (corn oil)	Control (corn oil + DMSO)	5 mg/kg	50 mg/kg	300 mg/kg	2000 mg/kg
AST (U/L)	197.6 ± 72.89	162.3 ± 46.69	164.1 ± 30.03	175.3 ± 98.03	158.2 ± 41.58	162.5 ± 9.78
ALT (U/L)	66.92 ± 14.34	68.64 ± 23.14	72.00 ± 13.68	79.42 ± 37.81	67.70 ± 10.77	48.80 ± 9.67
LDH (U/L)	683.8 ± 164.1	493.4 ± 114.2	644.2 ± 220.0	472.6 ± 174.1	390.2 ± 90.32	489.6 ± 179.1
GGT (U/L)	1.27 ± 0.47	1.06 ± 0.68	1.09 ± 0.46	1.86 ± 0.50	0.87 ± 0.15	0.36 ± 0.50
CHE (U/L)	315.4 ± 56.9	324.3 ± 82.1	453.1 ± 125.2	417.5 ± 98.58	492.5 ± 79.73	382.0 ± 85.15
Creatinine (mg/dL)	0.44 ± 0.03	0.49 ± 0.12	0.50 ± 0.07	0.50 ± 0.04	1.13 ± 0.64*	0.50 ± 0.03
Total protein (g/dL)	6.30 ± 0.99	5.87 ± 0.86	6.12 ± 0.38	5.45 ± 0.22	5.62 ± 0.78	5.18 ± 0.32
Albumin (g/dL)	2.04 ± 0.78	2.57 ± 0.73	2.06 ± 0.95	2.27 ± 0.53	2.45 ± 0.14	2.35 ± 0.66
Globulin (g/dL)	4.26 ± 0.92	3.31 ± 0.75	4.05 ± 0.85	3.19 ± 0.50	3.17 ± 0.69	2.83 ± 0.74
A/G	0.52 ± 0.29	0.81 ± 0.30	0.57 ± 0.37	0.77 ± 0.27	0.79 ± 0.14	0.90 ± 0.35

AST: aspartate aminotransferase; ALT: alanine aminotransferase; LDH: lactate dehydrogenase; GGT: gamma-glutamyltransferase; CHE: cholinesterase; A/G: albumin and globulin. *Significant difference with the control group (*P* < 0.05).

**Table 7:** Effect of the acute dermal exposure of LQM 996 on selected parameters (mean ± SD) in Wistar rats.

Parameter	Group				
	Control (corn oil)	Control (corn oil + DMSO)	500 mg/kg	2000 mg/kg	5000 mg/kg
AST (U/L)	201.5 ± 62.90	218.6 ± 79.04	212.3 ± 44.65	179.5 ± 37.17	207.8 ± 44.36
ALT (U/L)	85.87 ± 9.81	86.47 ± 17.24	87.88 ± 20.86	80.38 ± 8.56	77.54 ± 16.48
LDH (U/L)	732.0 ± 129.5	603.0 ± 172.1	719.6 ± 157.6	640.2 ± 116.6	1270 ± 652.2*
GGT (U/L)	1.01 ± 0.51	1.15 ± 1.62	0.29 ± 0.43	0.92 ± 0.90	1.06 ± 1.33
CHE (U/L)	457.5 ± 161.4	360.9 ± 66.32	435.3 ± 96.30	488.6 ± 157.8	546.4 ± 116.0
Creatinine (mg/dL)	0.51 ± 0.23	0.64 ± 0.21	0.57 ± 0.07	0.54 ± 0.01	0.54 ± 0.13
Total protein (g/dL)	6.59 ± 0.45	6.38 ± 0.26	6.59 ± 0.55	6.28 ± 0.76	6.62 ± 1.01
Albumin (g/dL)	2.53 ± 0.99	2.51 ± 0.82	2.25 ± 0.89	2.75 ± 0.83	2.87 ± 0.79
Globulin (g/dL)	4.06 ± 1.20	3.87 ± 1.03	4.34 ± 0.96	3.53 ± 0.47	3.74 ± 0.65
A/G	0.72 ± 0.41	0.73 ± 0.40	0.57 ± 0.34	0.80 ± 0.13	0.78 ± 0.22

AST: aspartate aminotransferase; ALT: alanine aminotransferase; LDH: lactate dehydrogenase; GGT: gamma-glutamyltransferase; CHE: cholinesterase; A/G: albumin and globulin. *Significant difference with the control group (*P* < 0.05).
